# Techniques for Thyroidectomy and Functional Neck Dissection

**DOI:** 10.3390/jcm13071914

**Published:** 2024-03-26

**Authors:** Orhan Agcaoglu, Serkan Sucu, Safa Toprak, Serdar Tezelman

**Affiliations:** Department of General Surgery, School of Medicine, Koç University, Istanbul 34450, Turkey; oagcaoglu@kuh.ku.edu.tr (O.A.); ssucu@kuh.ku.edu.tr (S.S.); satoprak@kuh.ku.edu.tr (S.T.)

**Keywords:** thyroidectomy, functional neck dissection, thyroid cancer, neck dissection

## Abstract

Thyroidectomy is a commonly performed surgery for thyroid cancer, Graves’ disease, and thyroid nodules. With the increasing incidence of thyroid cancer, understanding the anatomy and surgical techniques is crucial to ensure successful outcomes and minimize complications. This review discusses the anatomical considerations of the thyroid and neck, including lymphatic drainage and the structures at risk during thyroidectomy. Emphasis is placed on the significance of cautious dissection to preserve critical structures, such as the parathyroid glands and recurrent laryngeal nerve. Neck dissection is also explored, particularly in cases of lymph node metastasis, in which its proper execution is essential for better survival rates. Additionally, this review evaluates various thyroidectomy techniques, including minimally invasive approaches, highlighting their potential benefits and limitations. Continuous surgical knowledge and expertise updates are necessary to ensure the best results for patients undergoing thyroidectomy.

## 1. Introduction

Thyroidectomy is the most frequently performed surgery in endocrine surgery, which is commonly performed to treat thyroid diseases, such as thyroid cancer, benign thyroid nodules, or certain thyroid disorders, that do not respond to medical management. When performed by qualified surgeons, it is a safe and effective operation.

The American Thyroid Association (ATA) and National Comprehensive Cancer Network (NCCN) guidelines outline specific indications for thyroidectomy, emphasizing a comprehensive and patient-specific approach. Thyroidectomy is recommended for benign thyroid nodules that are large enough to cause compressive symptoms, such as difficulty breathing or swallowing [1]. Likewise, the thyroid gland should be removed in case of a significantly enlarged thyroid causing compressive symptoms or extending substernally. Moreover, thyroidectomy is indicated for patients with Graves’ disease who are unresponsive to antithyroid medications or radioactive iodine therapy. In cases of toxic nodular goiter, surgery on the thyroid gland may be warranted if hyperthyroidism persists despite treatments. Finally, thyroid cancer is the most definitive indication for thyroidectomy and neck dissection. The extent of the surgery is determined based on the cancer type, size, and stage and the presence of metastasis. For papillary and follicular thyroid carcinoma, the ATA guidelines recommend total thyroidectomy for tumors larger than 1 cm or in the presence of extrathyroidal extension, aggressive histological features, and lymph node metastasis [1,2]. However, the NCCN guidelines suggest that lobectomy is sufficient for intrathyroidal low-risk differentiated thyroid tumors up to 4 cm [3]. If tumors do not present high-risk features and are smaller than 1 cm, lobectomy might be appropriate for the treatment of microcarcinoma according to both the ATA and NCCN guidelines.

Additionally, therapeutic central and lateral lymph node dissection is only performed in cases in which there is clinical evidence of metastatic lymph nodes. In medullary thyroid cancer, total thyroidectomy with central lymph node dissection is performed for all patients because it quickly spreads into the central lymph node due to its aggressive tumor feature [1]. If the patient is diagnosed with familial syndromes, prophylactic thyroidectomy might be indicated based on the calcitonin levels and genetic tests. Overall, thyroidectomy and neck dissection indications should be determined with a multidisciplinary and patient-centered approach.

The thyroid cancer incidence increased from 4.9 per 100,000 in 1975 to 14.3 per 100,000 in 2009, attributed to the expanded use of neck ultrasonography in clinical practice [4,5]. Even though the increased use of neck ultrasonography has made it possible to diagnose thyroid malignancies early, in a significant portion of patients, the thyroid cancer metastasizes to the lymph nodes in the central and lateral neck [6]. Lymph node metastasis is important because it changes the management of thyroid cancer. Thyroidectomy only is enough for patients with small (T1,T2), noninvasive, and clinically node-negative PTC (papillary thyroid carcinoma) and for most follicular cancers, while patients with metastatic lymph nodes and T3 and T4 PTC require neck dissection [1]. The recurrence rate is high without anatomical neck dissection, but it also bears the increased risk of recurrent laryngeal nerve paralysis and hypoparathyroidism [7,8]. Therefore, the correct indication, an understanding of the anatomy, and careful dissection are vital.

## 2. Thyroid and Neck Anatomy

The thyroid gland is anterior to the trachea, extending from the fifth cervical vertebra to the first thoracic vertebra. It consists of the right and left lobes connected at the midline via the isthmus [9]. Occasionally a pyramidal lobe might extend superiorly. According to a study from Kim et al., the pyramidal lobe was present in 44% of the patient CT scans [10]. The thyroid receives its blood supply from the superior and inferior thyroidal arteries. The superior thyroid artery originates from the external carotid artery, and the inferior thyroid artery originates from the thyrocervical trunk. Venous drainage of the thyroid follows three separate pathways. The superior thyroid vein accompanies the superior thyroid artery and drains into the internal jugular vein, and the middle thyroid vein drains into the internal jugular vein. The inferior thyroid vein might drain into the internal jugular or the brachiocephalic trunk [9].

One of the most significant structures related to the thyroid gland is the recurrent laryngeal nerve (RLN), a branch of the vagus nerve. Recurrent laryngeal nerves innervate the intrinsic muscles of the larynx and provide sensory innervation to the glottic larynx [11]. The right RLN loops posteriorly to the subclavian artery, while the left RLN loops posteriorly to the arch of the aorta [9]. The right RLN nerve reaches from the tracheoesophageal groove near the inferior to the thyroid gland, and the left RLN ascends to the larynx in the tracheoesophageal groove [9]. The anatomical landmarks for identifying the RLN include the Berry ligament and Zuckerkandl tubercle [11]. In addition, another nerve that has a close relationship with the thyroid gland and is therefore at risk for injury, especially in superior pole dissection, is the external branch of the superior laryngeal nerve [11]. It descends anteriorly and medially with a superior thyroid artery from its course with the pharyngeal constrictor muscle and innervates the cricothyroid muscle [12]. Injury to this nerve might also damage the voice quality and strength [12].

Regional lymph node spread from thyroid cancer can be classified as lateral and central neck metastases [6]. Neck dissection is mostly required when there is metastatic spread to the lymph nodes, and it provides a better survival rate and less recurrence [13]. However, radical neck dissection is considered too aggressive because of the increased complication rate. For this reason, functional neck dissection was defined by Suarez et al. in 1962 [14,15]. Functional neck dissection includes the removal of lymph nodes on metastatic sites following fascial planes while preserving nonlymphatic structures, including the sternocleidomastoid, digastric, and stylohyoid muscles, internal jugular vein, spinal accessory, and great auricular nerves [15]. Although it provides a lower complication rate than radical neck dissection, it is a technically challenging operation requiring thorough knowledge of neck anatomy [8,16].

Lymphatic drainage of the thyroid gland can be divided into major and minor drainage. Major drainage occurs in the middle, lower jugular, and posterior triangle nodes, while minor drainage occurs in the pretracheal, paratracheal, and superior mediastinal lymph nodes. The neck is divided into central and lateral compartments. The lateral compartment is composed of level I (submandibular and submental), level II (upper jugular), level III (middle jugular), level IV (lower jugular), and level V (posterior triangle) lymph nodes. In contrast, the central compartment of the neck is composed of level VI (prelaryngeal, pretracheal, and right and left paratracheal) lymph nodes [6,17].

## 3. Preoperative Planning of Thyroidectomy and Neck Dissection

Before performing a thyroidectomy, medical professionals usually employ a systematic assessment procedure to determine the necessity, extent, and strategy of the operation. A comprehensive medical history that emphasizes the symptoms, the thyroid disease risk factors, prior neck radiation, and any family history of thyroid diseases are all part of the first evaluation. The thyroid gland’s size, consistency, and shape are evaluated, as well as any palpable nodules, during the physical examination. Afterward, the thyroid function must be evaluated with tests like Free T4, TSH (thyroid-stimulating hormone), and others, especially in the context of nodules and planned surgery [1]. Furthermore, many centers have established a routine practice of measuring the calcitonin levels from plasma in conjunction with performing a biopsy upon the detection of a suspicious thyroid nodule. Moreover, the main imaging technique for assessing thyroid nodules and directing fine-needle aspiration cytology (FNAC) is ultrasonography. It assists in determining the type, number, and features of the thyroid nodules and any involvement of the lymph nodes. In the preoperative assessment for thyroidectomy, FNAC is an effective technique, particularly for nodules that exhibit clinical or ultrasonographic characteristics that raise suspicions of malignancy [18]. Furthermore, regions of the thyroid gland that are hyperactive or underactive can be found using radioactive thyroid scanning in situations of hyperthyroidism. Locally invasive thyroid cancer is evaluated with Computed Tomography (CT) or Magnetic Resonance Imaging (MRI) to determine the extent and invasiveness of the thyroid tumors [18]. As advised by a multidisciplinary team and the ATA recommendations, the use of different imaging modalities in the treatment of thyroid cancer should be tailored to the unique clinical circumstances of each patient.

### 3.1. Ultrasonography (USG)

The primary imaging modality for the first assessment of thyroid nodules is ultrasound, which is also essential for treating thyroid cancer. USG is a sensitive imaging modality in the context of detecting thyroid nodules, assessing their characteristics, and guiding fine-needle aspiration biopsies. The size, content, echogenicity, and presence of suspicious characteristics, such as microcalcifications, irregular margins, and enhanced vascularity, which may point to malignancy, can all be assessed by ultrasound [19].

### 3.2. Computed Tomography (CT)

The ATA guidelines recommend the use of CT imaging for thyroid cancer with extrathyroidal extension or the involvement of important adjacent tissues, like the trachea, esophagus, or major blood arteries [20]. For surgical planning and the assessment of large goiters or substernal expansions, CT can offer comprehensive anatomical information [21]. Furthermore, CT can be helpful in staging patients with advanced differentiated thyroid carcinoma by detecting distant metastases, particularly in the lungs, which are a common site of metastasis [22]. However, non-contrast-enhanced CT scans are the superior choice in these circumstances because iodine-based contrast agents should be used with caution, as they may interfere with later radioactive iodine treatments [23]. Moreover, particularly in individuals with recurrent or chronic illness, CT can be helpful in combination with other imaging modalities, such as ultrasonography and radioactive iodine scans, to identify structural diseases that may not be seen in different imaging modalities.

### 3.3. Magnetic Resonance Imaging (MRI)

MRI enables the determination of the extent of invasive thyroid cancers and allows for differentiating between the cancerous tissue and surrounding structures, like muscles, the trachea, the esophagus, and vascular structures, due to a better soft-tissue contrast compared to CT [20]. Patients with severe or recurring illnesses benefit the most from MRI because it provides comprehensive pictures without requiring the use of an iodine-based contrast, which can interfere with upcoming radioactive iodine treatments. MRI can evaluate metastatic lesions at specific sites, such as the brain or spinal cord, where its superior soft-tissue contrast allows for a more precise delineation of the disease extent [22]. However, the guidelines advise against using MRI routinely for the first examination of thyroid nodules because of its high sensitivity and low specificity, which may result in unnecessary examinations. Overall, it is an essential tool for comprehensive preoperative planning, the assessment of recurrent or residual cancer tissue in the thyroid bed or lymph nodes, and the demarcation of tumor boundaries and vascular penetration.

### 3.4. Positron Emission Tomography–Computed Tomography (PET-CT)

PET-CT is not commonly utilized for the preliminary evaluation of thyroid nodules. It is recommended for patients with differentiated thyroid carcinoma who have increased thyroglobulin levels but negative radioactive iodine scans in order to assess possible distant metastases [24]. Furthermore, anaplastic and poorly differentiated thyroid tumors are types of more aggressive thyroid cancers for which PET-CT is helpful in the management of the disease because it can offer crucial information about the disease’s extent and possible sites of metastasis [25]. Furthermore, PET-CT can be helpful in examining suspected recurring diseases and can help detect malignancies that may not be seen with conventional imaging modalities [26].

## 4. Techniques for Thyroidectomy

Thyroidectomy is a surgical procedure to remove all or parts of the thyroid gland. Kebebew and Clark proposed a classification system based on the extent of the surgery: lumpectomy refers to a neoplasm that is removed with minimal thyroid tissue; partial thyroidectomy is the removal of a neoplasm surrounded with healthy thyroid tissue; subtotal thyroidectomy refers to the removal of more than half of each lobe and the isthmus and to the bilateral removal of more than 50% of each lobe with the isthmus; lobectomy, also called hemithyroidectomy, refers to totally removing one lobe of the thyroid gland with the isthmus [27]; near-total thyroidectomy is the removal of one lobe and the isthmus with less than 5% of the contralateral lobe remaining; total thyroidectomy is the removal of both lobes and the isthmus with the preservation of the parathyroid glands and recurrent laryngeal nerves [27].

The success of a thyroid operation depends on the surgeons’ understanding of the anatomy, dissection technique, and steps of the procedure. The main anatomical steps include the exposure of the thyroid, dissection of the upper pole and superior laryngeal nerve, dissection of the lateral part of the thyroid lobe, preservation of the recurrent laryngeal nerve and parathyroid glands, and closure [28].

To date, neuromonitorization has played a crucial role in thyroidectomies by providing valuable information and guidance for the identification and dissection of the laryngeal nerves. Furthermore, neuromonitorization is essential in cases with anatomical variations and challenging anatomies because of the increased risk of nerve injury [29]. Over the last two decades, continuous- and intermittent-neuromonitoring systems have been developed, and these devices detect the response of the laryngeal muscles to recurrent laryngeal nerve stimulation and record them as electromyographic signals [9].

**Figure 1:**
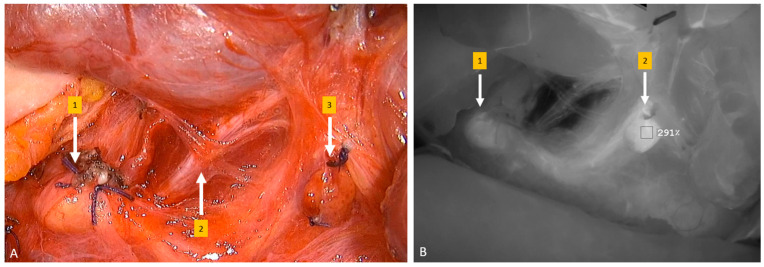
(**A**) Intraoperative image of left parathyroid glands and left inferior (recurrent) laryngeal nerve (1—left inferior parathyroid gland, 2—left recurrent laryngeal nerve, 3—left superior parathyroid gland); (**B**) intraoperative fluorescence image using SPY-PHI (Stryker Corp., Kalamazoo, MI, USA) showing quantitative increase in perfusion of left parathyroid glands after intravenous ICG (Verdye^TM^, Diagnostic Green Ltd., Athlone, Ireland) injection (1—left inferior parathyroid gland, 2—left superior parathyroid gland).

Another important development regarding thyroid surgery is fluorescent imaging, which emerged as a useful tool in various kinds of surgical fields. Its reliability has been proven in various studies for its potential use in thyroid surgery. It is utilized to identify, visualize, and preserve the parathyroid glands in order to prevent post-thyroidectomy hypoparathyroidism, which is a common complication of thyroidectomy, especially with lymph node dissection. There are two fluorescence imaging approaches: contrast-enhanced fluorescence and autofluorescence. An exogenous contrast agent is administrated as intravascular or local contrast-enhanced fluorescence, while the intrinsic florescent properties of the thyroid and parathyroid are visualized via specific optical modalities in autofluorescence [30]. Indocyanine green (ICG) is the most commonly used agent in thyroid surgery and is applied intravenously. Moreover, the vascularization of the parathyroid glands can be easily evaluated intraoperatively by visualizing ICG, and thus the risk for parathyroid damage can be minimized [31,32] (Figure 1).

### 4.1. Technical Details of a Conventional Thyroid Surgery

The patient is placed in a supine position with their arms at their side and their neck extended. A towel is placed under the neck, which provides adequate exposure by extending the neck. A transverse incision, defined as a Kocher incision, is made following a natural skin crease marked preoperatively. The level of the incision (mostly the natural skin fold) is crucial for optimal cosmetic results, and it should be placed neither too cephalad nor too caudal [28]. The incision is deepened, and the subplatysmal flaps from the sternal notch to the thyroid cartilage are elevated. Following the raphe, the strap muscles are split along the midline.

Dissection is continued to the lateral space between the thyroid gland and the carotid sheath; the middle thyroid vein is divided in this space [28]. The pyramidal lobe must be dissected off from the trachea if present. The superior lobe of the thyroid gland is mobilized and dissected from its attachments, and the superior thyroidal artery is ligated. When the superior lobe is dissected, the external branch of the superior laryngeal nerve must be protected, as it runs adjacent to the superior thyroidal artery and damage to it could result in hoarseness [33]. The complete division of the superior vessels and the superior pole allows for the anteromedial mobilization of the thyroid gland [9]. The medial and inferior parts of the thyroid are dissected according to the capsular dissection technique, in which dissection is carried out closely, separating the parathyroid glands with their vascular pedicles from the thyroid surface, and the inferior thyroid artery is divided close to the thyroid gland with the minimal exposure of the recurrent laryngeal nerve [34]. Following lateral dissection, the thyroid gland is elevated from the trachea, and the same procedure is applied to the contralateral side. Careful hemostasis is applied, and the Valsalva maneuver is performed at the end of the surgery to detect possible bleeding vessels. Strap muscles and the platysma are reapproximated with absorbable sutures. The skin is closed subcutaneously with a monofilament absorbable suture. Routine drain application is unnecessary after thyroidectomy, but it can be placed if the removed thyroid gland is large or according to clinical suspicion [35].

### 4.2. Vessel-Sealing Energy Devices

Energy devices play an essential role in evolved thyroidectomy and neck dissection alongside developed surgical techniques. These devices are utilized for the cutting, coagulation, sealing, and approximation of tissue. Energy devices used in thyroidectomy are categorized into various groups, such as the ultrasonic coagulation–dissection system [HARMONIC FOCUS™ (Ethicon, Cincinnati, OH, USA)], the electrothermal bipolar vessel-sealing system [LigaSure™ Exact Dissector (Medtronic, Minneapolis, MN, USA)], and the ultrasonic–electrothermal hybrid coagulation–dissection system [THUNDERBEAT Open Fine Jaw (Olympus Medical Systems Corp., Tokyo, Japan)]. The ultrasonic coagulation–dissection system uses frequencies between 23 and 55 kHz to produce mechanical cutting, desiccation, protein coagulation, cavitation, or a combination of these effects. These devices have an ultrasonic transducer that vibrates at a high frequency, allowing for the rapid cutting of the tissue with less thermal spread [36]. The study on utilizing the Harmonic Scalpel for neck dissections indicates that patients undergoing surgery with the ultrasonic device experienced reduced blood losses and lower drainage volumes, alongside decreased surgery durations, compared to those who underwent surgery using traditional knot-tying techniques [26]. Moreover, the electrothermal bipolar vessel-sealing system combines a precise amount of bipolar electrocoagulation with pressure on the tissue and denatures the collagen and elastin in the vessel walls, thereby leading to effective vessel sealing [37]. These devices incorporate a tissue-based feedback program that ensures that the right amount of energy is delivered to minimize surrounding tissue damage. A study assessing the effectiveness of the electrothermal bipolar vessel-sealing system in total thyroidectomy demonstrates a remarkable reduction in the operative time by 14 min, without significant differences in the postoperative complications and hospital stay [38]. Furthermore, the ultrasonic–electrothermal hybrid coagulation–dissection system combines ultrasonic energy for cutting and dissection with bipolar energy for the efficient coagulation and sealing of the vessels. It integrates bipolar heat energy laterally with ultrasonic energy centrally, facilitating sealing capabilities even in large vessels [39]. The system provides precise cutting, reduced blood loss, and enhanced surgical outcomes by combining the benefits of both ultrasonic and electrothermal technologies. The study comparing Thunderbeat with standard electrocautery devices shows that ultrasonic–electrothermal hybrid devices lead to a significant reduction in blood loss (from 431 mL to 210 mL, *p* = 0.046) and a decrease in the operative time (from 150 min to 101 min, *p* = 0.014) without an increase in the complication rates [40]. Overall, the use of energy devices in surgery has had a significant impact on patient care, the operative times, and the minimization of complications. These devices have enhanced surgical efficiency, improved outcomes, and contribute to the advancement of thyroid surgery.

## 5. Neck Dissection Techniques and the Concept of Functional Neck Dissection

Cervical lymphadenectomy holds a crucial position in treating thyroid malignancy. Removing cervical lymph nodes, whether identified through clinical examination/radiographic imaging (therapeutic lymphadenectomy) or not (prophylactic lymphadenectomy), can potentially decrease the recurrence rates and enhance the disease-specific survival in certain forms of thyroid cancer [41].

An additional topographical classification that has gained widespread acceptance, introduced by K. Thomas Robbins in 1991 and updated by him in 2001, has had a significant impact on conventional oncological practice [42,43]. This classification system aims to establish consistency in naming different types of cervical lymph node dissections by categorizing the involved topographical regions and any anatomical structures sacrificed during the procedure. Each nodal level is not indicative of separate lymphatic drainage areas; instead, they are categorized based on specific anatomic landmarks. This classification helps the surgeon minimize injury to associated structures during compartment-specific lymphadenectomy procedures. Consequently, the neck is divided into six levels (five on each side, plus a sixth anterior median level).

Over a century ago, George Crile initiated the idea of neck dissection as an essential addition to the treatment of upper-aerodigestive-tract tumors [44]. The most common classification for cervical lymphadenectomy is based on the specific anatomical area sampled. In the past, neck dissection was commonly carried out using an extensive technique. Among these classifications, radical and modified radical neck dissections are the most extensively described nodal-harvesting techniques for thyroid cancer.

The concept of functional neck dissection was proposed by Suarez et al. for laryngeal cancer in order to protect the function while providing proper oncological lymph node dissection [45]. The term “Functional Neck Dissection” is often confused with modified radical neck dissection, but it is a concept rather than a synonym for a type of neck dissection [46]. This concept includes dissecting along the fascial planes to remove lymph nodes and preserve nonlymphatic structures regardless of the anatomical boundaries, while modified radical neck dissection implies removing lymphatic tissue while preserving nonlymphatic tissue as much as possible [15,42,45]. Thus, it is key to differentiate the two terms in order to understand the concept of functional neck dissection. Moreover, the morbidity is significantly decreased with the introduction of functional neck dissection [15]. Therefore, utilizing this new method, the surgeon can maintain a similar level of radicality as in Crile’s proposed neck dissection while safeguarding crucial structures, like the sternocleidomastoid muscle, internal jugular vein, and spinal accessory nerve, as long as the lymph node capsule remains intact.

Even though conservational techniques improved the morbidity significantly, the lack of standardization of new techniques led to confusion and a lack of uniformity. To overcome problems in classification, Robbins et al. suggested a level system for neck anatomy and the classification of neck dissection in 1991 that includes radical neck dissection, modified radical neck dissection, selective neck dissection, and extended radical neck dissection [47]. Furthermore, they considered radical neck dissection, which is defined as the en-bloc removal of lymphatic structures with the sternocleidomastoid muscle, internal jugular vein, and accessory nerve, as the classical procedure for cervical lymphadenectomy; the preservation of nonlymphatic structures during dissection as modified radical neck dissection; the preservation of one or more lymph node groups as selective neck dissection; and the addition of other lymph node groups or nonlymphatic entities to radical neck dissection as extended radical neck dissection [47]. In 2002, the committee for neck dissection classification reviewed the system proposed in 1991. They decided to continue the use of the level system to classify the location of lymph node disease and introduced a new sublevel system [43]. For the neck dissection, they continued using the terminology of radical, extended radical, and modified radical neck dissection but redefined and classified selective neck dissection according to the malignancy type [43] (Table 1). Furthermore, selective lymph node dissection for thyroid cancer encompasses both central and lateral neck dissection.

### 5.1. Central Neck Dissection

The area bordered by the hyoid bone superiorly, clavicles and sternum inferiorly, and carotid arteries laterally is defined as the central region, including only the level VI neck compartment. The central region contains prelaryngeal, pretracheal, and right and left paratracheal lymph nodes [17].

Central neck dissection in thyroid cancer can be performed for prophylactic and therapeutic reasons. Therapeutic dissection is performed when there are metastatic lymph nodes detected via clinical or radiological examination in the central region, and prophylactic dissection is performed when there are no obvious metastatic lymph nodes [17]. Central neck dissection is not indicated for T1 and T2 tumors because of the lower risk of metastasis, but when the tumor is larger in size (T3, T4), multicentric, an aggressive subtype, or the patient is young or has BRAF mutations, the risk of metastasis to the central lymph nodes is high [48,49].

**Figure 2:**
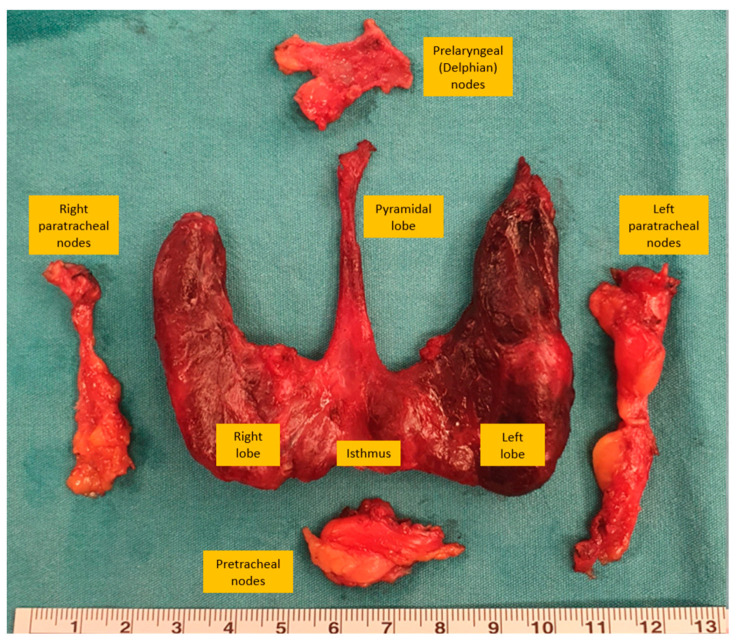
Bilateral total thyroidectomy with central-compartment (level 6) dissection specimen.

During central dissection, the prelaryngeal lymph nodes are excised superiorly, the pretracheal lymph nodes are removed inferior to the innominate artery, and the paratracheal lymph nodes are removed inferior to the cricoid cartilage [17]. Preservation of the ipsilateral recurrent laryngeal nerve and parathyroid glands is crucial during central neck dissection. Parathyroid glands receive their blood supply from the inferior thyroid artery, and care must be taken to preserve the branch of the inferior thyroid artery entering the parathyroid gland by ligating it close to its branch entering the thyroid capsule to prevent the devascularization of the parathyroid gland [50]. When the parathyroid gland loses its blood supply, autologous transplantation can be performed, but a biopsy is usually required to avoid the implantation of metastatic lymph nodes (Figure 2).

### 5.2. Selective Neck Dissection

Selective neck dissection was first classified as supraomohyoid, lateral, posterolateral, and anterior in 1991 [47]. However, with the recognition of distinctions in the spread pattern, this anatomical classification system was changed to a new system based on the spread pattern of the tumor types [43]. The first proposed selective lymph node dissections in 2002 included the dissection of regions I–III for oral cavity cancers and regions II–IV for laryngeal cancers [43]. For thyroid cancer, the regions for selective neck dissection depend on the primary tumor, its extent, and the pattern of the metastatic spread. The preoperative assessment of the anterior and lateral neck compartments for metastasis, through clinical examination or imaging studies, is a major determinant of the extent of the selective neck dissection, especially in cases of papillary thyroid cancer, due to its potential for unusual metastatic behavior, such as skip metastases [51].

### 5.3. Lateral Neck Dissection

Treatment for thyroid cancer includes the dissection of the lymph nodes in the lateral neck compartments when a metastatic lymph node in this section is present [52]. Metastasis can be obvious in a physical examination or detected in neck ultrasound. Furthermore, ultrasound is valuable not only for detecting nonpalpable lymph nodes but it also helps to guide the extent of the lymphadenectomy [53]. The key neck areas dissected during lateral dissection include level III, level IV, the anterior aspect of level V, and level II if positive lymph nodes are suspected [54]. However, there is debate about the effectiveness of level I and V dissection because papillary thyroid cancer rarely metastasizes to these areas [55]. Moreover, Neiderman et al. also found that there was no significant difference between patients who underwent level V node dissection compared to those who did not [56]. As a result, it might not be necessary to perform lymph node dissection on level I and V in all cases.

**Figure 3:**
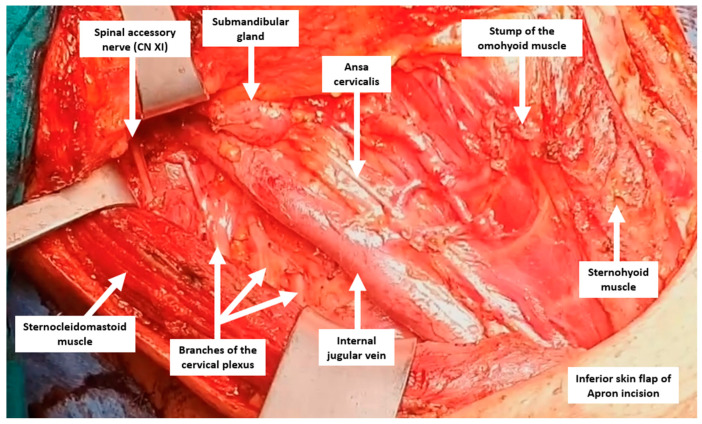
Intraoperative image of a patient who underwent total thyroidectomy, central neck (level 6), and left functional neck (level 2–3–4–5) dissection.

**Figure 4:**
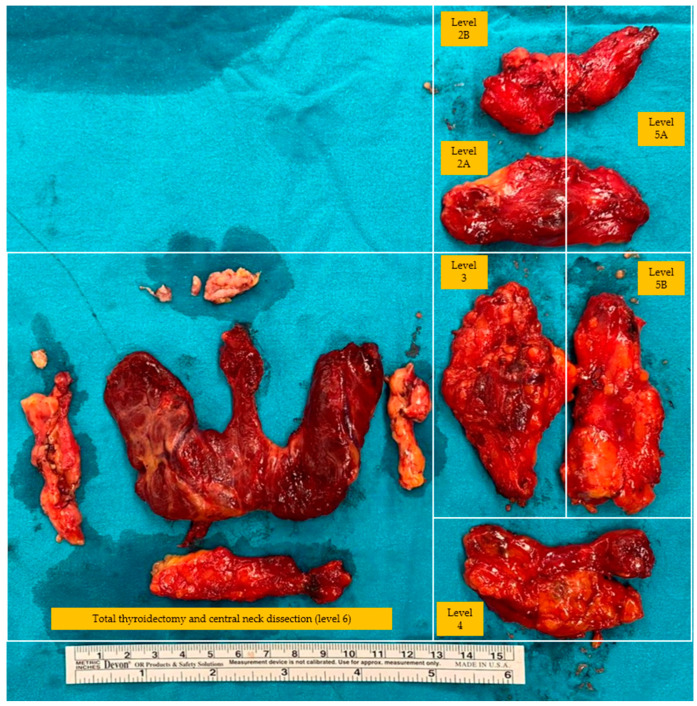
Bilateral total thyroidectomy with central neck dissection and left functional lateral neck dissection specimen.

The extent of the incision depends on whether a thyroidectomy is performed in the same procedure. If a thyroidectomy is also performed, the Kocher incision can be extended laterally to include the affected side of the neck, and if not, a transverse incision can be made at the level of the lower edge of the cricoid cartilage from the edge of the trachea to the anterior border of the trapezius muscle [57]. Subplatysmal flaps are made and extended medially up to the thyroid cartilage and superiorly up to the anterior to the carotid artery and internal jugular vein [54]. The anterior border of the sternocleidomastoid muscle is dissected, and the dissection plane is developed between the sternocleidomastoid muscle and strap muscles. The omohyoid muscle can be ligated and transected when encountered. Lymph nodes are anterior to the internal jugular vein, and the carotid artery can be dissected at this stage or at later stages of the surgery [54]. The carotid sheath is opened, and the lateral border of the internal jugular vein is dissected. The soft tissue containing lateral lymph nodes is exposed when the internal jugular vein is retracted medially. The phrenic nerve can be found on the anterior scalene muscles at the inferior border of this lymphatic tissue located posterior to the transverse cervical artery and can be sacrificed, if necessary, during dissection. Dissection is continued inferiorly along the lateral border of the internal jugular vein while identifying and preserving the carotid artery and vagus nerve. The horacic duct can be encountered looping behind the internal jugular vein if the dissection is performed on the left side. Soft tissue, including the lymph node package, is dissected off the anterior scalene muscles and elevated anteriorly. The specimen is retracted anteromedially, and the cervical plexus inferiorly and spinal accessory nerve superiorly are preserved during the dissection of the specimen. The lymph nodes inferior and medial to the spinal accessory nerve are included in the specimen, while the contents superior and medial are not included [50] (Figure 3 and Figure 4). A suction drain is routinely placed for a possible lymphatic leakage and the faster adhesion of the flaps through negative pressure. The platysma is closed with an interrupted-polyfilament absorbable suture, and the skin is closed with an absorbable monofilament suture. When the drainage output sufficiently decreases, the drain is removed. Specifically, the drain is generally kept in the thyroid lodge for an average of three days in cases of lateral lymph node dissection [50].

## 6. Minimally Invasive Operative Techniques

Minimally invasive thyroidectomy techniques have been developed to improve the recovery time, tissue trauma, and cosmetic results. Minimally invasive techniques include robotic, endoscopic, and video-assisted techniques using various approaches, such as the cervical, axillary, breast, post-auricular, or transoral approaches [58]. Among the approaches mentioned, the most commonly used ones include the endoscopic and robotic transoral vestibular and transaxillary approaches [58]. Even though minimally invasive thyroidectomies provide improved cosmesis, they also have limitations. The major limitations include an increase in the operative time, increased transient recurrent laryngeal nerve paralysis, and the creation of wide spaces during flap elevation. Furthermore, it requires pronounced learning curves and higher costs [59].

### 6.1. Minimally Invasive Video-Assisted Thyroidectomy (MIVAT)

Minimally Invasive Video-Assisted Thyroidectomy (MIVAT) first emerged in 1999, aimed at offering a less invasive option for thyroid surgery. It provides benefits such as smaller incisions, reduced postoperative pain, and superior cosmetic outcomes compared to standard open thyroidectomy while maintaining comparable oncologic radicality, time costs, and complication rates [60,61]. The criteria for MIVAT include a nodule size of less than 35 mm and a total thyroid volume of less than 25 cc, while severe thyroiditis and suspicious metastatic lymph nodes in the lateral neck are contraindications. The procedure involves a small 1.5 cm incision above the sternal notch and employs a 5 mm, 30° endoscopic lens, focusing on the medial retraction of the thyroid lobe to prevent recurrent laryngeal nerve damage, using the tuberculum of Zuckerkandl as a crucial landmark [60]. Moreover, complications of this procedure include recurrent laryngeal nerve palsy and temporary hypoparathyroidism. A study from 1998 to 2019 on 763 patients confirms MIVAT’s safety, showing a 7% transient hypoparathyroidism rate and a 1.4% permanent recurrent laryngeal nerve palsy rate [62]. Furthermore, a comparative study found that MIVAT had shorter operation times, less postoperative pain, and higher cosmetic satisfaction compared to the conventional thyroidectomy, with no significant difference in the rates of transient recurrent laryngeal nerve palsy or hypoparathyroidism [61]. Overall, MIVAT is a feasible and safe procedure that offers cosmetic benefits without increasing major complications, but it requires careful patient selection, is appropriate for only a limited number of patients, and necessitates a considerable learning curve.

### 6.2. Endoscopic Thyroidectomy

Endoscopic thyroidectomy, which was first performed by Ikeda in 1997, offers a minimally invasive alternative to open thyroidectomy [63]. It offers better cosmetic outcomes, a lower rate of surgical complications, and less postoperative pain compared to the conventional thyroidectomy [64]. In this regard, various techniques, such as the gasless unilateral transaxillary, bilateral axillo-breast, and transoral vestibular (TOETVA) approaches are established based on the patient needs and the anatomical limitations of the endoscopic access. These techniques underscore reducing the operative time, controlling bleeding, and ensuring lymph node assessment, as well as less postoperative pain [65]. Therefore, endoscopic thyroidectomy has expanded its indications from benign thyroid diseases to malignant conditions with increasing endoscopic surgical experiences. The study reviewing the outcomes of the TOETVA in 132 thyroid cancer patients underscores that it is a safe and efficient procedure with an 87 min mean operation time, shorter hospitalization period, and low complication rate, including transient hoarseness and hypoparathyroidism [66]. To obtain the optimal outcomes and reduce the complication risk in thyroid cancer, candidates for endoscopic thyroidectomy should be selected properly based on the following criteria: the absence of metastasis, size of the thyroid gland, a nodule size suitable for endoscopic management, and no prior neck surgery or radiation therapy. Consequently, despite its significant advantages in terms of the aesthetic outcomes and reduction in postoperative complications, endoscopic thyroidectomy is not feasible for all patients with thyroid cancer.

### 6.3. Robotic Thyroidectomy

Robotic thyroidectomy, introduced by Chung in 2007, represents a significant advancement in the field of endocrine surgery, offering a minimally invasive alternative to conventional thyroidectomy in order to enhance the surgical precision, minimize scarring, and potentially reduce the recovery times [67]. Despite its advantages, robotic thyroidectomy has longer operative times, higher costs, and limited suitability for advanced thyroid cancer, so patient selection is critical for robotic thyroidectomy [68]. Patient selection requires careful consideration of the nodule size and location, patient comorbidities, and anatomical factors [69]. Ideal candidates have small, non-aggressive nodules, while there is no significant scar tissue secondary to previous neck surgery. However, robotic thyroidectomy is not suitable for individuals with advanced cancer, extensive local invasion, or lymph node metastasis [69]. Additionally, patients with inflammatory thyroid conditions or extensive neck surgery histories constitute higher complication risks due to the altered anatomy and dense scar tissue.

Robotic technology is successfully utilized for thyroid surgery in experienced centers in a manner similar to that of endoscopic techniques. Transoral robotic thyroidectomy (TORT), robotic thyroidectomy, and bilateral axillary breast-approach robotic thyroidectomy (BABA RT) are the mostly applied techniques in robotic thyroidectomy, as well as in endoscopic thyroidectomy. Furthermore, the procedure of robotic thyroidectomy involves creating a working space, docking the robot, and performing the surgery through the console. The surgical details vary depending on the approach, which are the transaxillary, transoral, retroauricular, and bilateral axillary breast (BABA) approaches [68]. The approach of the procedure is chosen based on the patient anatomy, the extent of the disease, and cosmetic preferences. In recent times, the robotic thyroidectomy technique that has been predominantly favored and implemented is the bilateral axillo-breast approach [70]. A study including 500 patients undergoing the robotic BABA revealed that the rate of transient vocal cord palsy and hypoparathyroidism is significantly lower compared to open thyroidectomy [70]. Moreover, the BABA has emerged as a more conducive technique for total thyroidectomies and the excision of large goiters compared to the transaxillary approach, which is attributed to its enhanced anatomical accessibility and the precision facilitating the comprehensive removal of the thyroid gland [71,72]. Ultimately, robotic thyroidectomy provides enhanced visualization and precision, leading to less complications, while it is also associated with a pronounced learning curve, extended durations of operation, and potentially higher costs.

## 7. Conclusions

Thyroidectomy is an essential surgical procedure for treating thyroid diseases, including cancer and nodules. Proper management and thorough knowledge of the anatomy and surgical technique are required in order to protect vital structures, such as the recurrent laryngeal nerve and parathyroid glands, especially in cases with lymph node metastasis, which requires neck dissection. While minimally invasive thyroidectomy techniques offer potential benefits, they come with their own challenges. As medical knowledge and surgical techniques continue to advance, maintaining expertise and staying updated is vital to ensure optimal patient outcomes and well-being.

Due to the extensive comprehension of the subject and the significant chronological variation in surgical techniques, examining all the surgical methods in a single article is quite challenging. Therefore, we aimed to discuss thyroidectomy and functional neck dissection by describing its general outlines and explaining the most commonly used techniques as comprehensively as possible.

## Figures and Tables

**Table 1:** Classification of neck dissection.

Type of Neck Dissection	Description
Radical Neck Dissection	Excision of all ipsilateral cervical lymph node clusters (level I–level V). In addition, the spinal accessory nerve, internal jugular vein, and sternocleidomastoid muscle are excised.
Modified Radical Neck Dissection	Removal of all lymph nodes typically excised during the radical neck dissection with the retention of one or more nonlymphatic structures (i.e., the spinal accessory nerve, internal jugular vein, sternocleidomastoid muscle).
Selective Neck Dissection	Indicates a cervical lymphadenectomy in which there is the preservation of one or more of the lymph node clusters that are commonly excised in radical neck dissection.
Extended Neck Dissection	Refers to the excision of one or more additional lymph node clusters or nonlymphatic entities, or both, that are not covered by the radical neck dissection (examples of these lymph node clusters include parapharyngeal, superior mediastinal, perifacial, paratracheal; examples of nonlymphatic entities include the carotid artery, hypoglossal nerve, vagus nerve, and paraspinal muscles).

Adapted from 2002 merican Head and Neck Society and American Academy of Otolaryngology–Head and Neck Surgery guidlines [43].

## Data Availability

Data sharing not applicable.
